# Conservation of Native Wild Ivory-White Olives from the MEDES Islands Natural Reserve to Maintain Virgin Olive Oil Diversity

**DOI:** 10.3390/antiox9101009

**Published:** 2020-10-17

**Authors:** Anallely López-Yerena, Antònia Ninot, Julián Lozano-Castellón, Elvira Escribano-Ferrer, Agustí J. Romero-Aroca, Angjelina Belaj, Anna Vallverdú-Queralt, Rosa M. Lamuela-Raventós

**Affiliations:** 1 Nutrition, Food Science and Gastronomy Department, XaRTA, Institute of Nutrition and Food Safety (INSA-UB), Pharmacy and Food Sciences School, University of Barcelona, 08028 Barcelona, Spain; naye.yerena@gmail.com (A.L.-Y.); jullozcas@gmail.com (J.L.-C.); avallverdu@ub.edu (A.V.-Q.); 2 Institute of Agrifood Research and Technology (IRTA), Fruit Science Program, Olive Growing and Oil Technology research team, 43120 Constantí, Spain; Antonia.Ninot@irta.cat (A.N.); Agusti.Romero@irta.cat (A.J.R.-A.); 3 CIBER Physiopathology of Obesity and Nutrition (CIBEROBN), Institute of Health Carlos III, 28029 Madrid, Spain; eescribano@ub.edu; 4 Department of Pharmacy and Pharmaceutical Technology and Physical Chemistry, Biopharmaceutics and Pharmacokinetics Unit, Institute of Nanoscience and Nanotechnology (IN2UB), Pharmacy and Food Sciences School, University of Barcelona, 08028 Barcelona, Spain; 5 Instituto de Investigación y Formación Agraria y Pesquera (IFAPA)—Centro “Alameda del Obispo”, Avda. Menéndez Pidal s/n, E-14004 Córdoba, Spain; angjelina.belaj@juntadeandalucia.es

**Keywords:** conservation, food, oleaster, plant diversity, polyphenol, fatty acid profile, sensorial analysis

## Abstract

Food diversity, and in particular genetic diversity, is being lost at an alarming rate. Protection of natural areas is crucial to safeguard the world’s threatened species. The Medes Islands (MI), located in the northwest Mediterranean Sea, are a protected natural reserve. Wild olive trees also known as oleasters make up part of the vegetation of the Meda Gran island. Among them, in 2012, a wild albino ivory-white olive tree with fruit was identified. Fruits were collected from this tree and their seeds were first sown in a greenhouse and then planted in an orchard for purposes of ex situ preservation. Seven out of the 78 seedling trees obtained (12%) produced ivory-white fruits. In autumn 2018, fruits from these trees were sampled. Although the fruits had low oil content, virgin olive oil with unique sensory, physicochemical, and stability characteristics was produced. With respect to the polyphenols content, oleacein was the main compound identified (373.29 ± 72.02 mg/kg) and the oleocanthal was the second most abundant phenolic compound (204.84 ± 52.58 mg/kg). Regarding pigments, samples were characterized by an intense yellow color, with 12.5 ± 4.6 mg/kg of chlorophyll and 9.2 ± 3.3 mg/kg of carotenoids. Finally, oleic acid was the main fatty acid identified. This study explored the resources of the natural habitat of the MI as a means of enrichment of olive oil diversity and authenticity of this traditional Mediterranean food

## 1. Introduction

Despite increasing recognition of the importance of biodiversity of natural and human communities and the tremendous conservation strategies aimed at detaining the decline in wild habitats at regional and national levels, biodiversity is rapidly diminishing worldwide [1,2]. The protection of natural areas is crucial to safeguard threatened species and represents the fundamental building blocks of most national and international conservation strategies [3]. Until 2018, 15 national parks, 152 natural parks, 291 nature reserves, 342 monuments natural, 57 protected landscapes, and more than 800 spaces have been declared as natural protected areas in Spain. In this sense, the Natura 2000 network protects 27% of the land area and about 13% of the waters in Spain [4]. The Medes Islands (MI) located in the northwest Mediterranean Sea were declared in 2010 a National Protected Natural Park for the great biodiversity of marine fauna and flora of the islands. The MI vegetation has been adapted to a series of abiotic (climate, wind, salinity, geology) and biotic factors. Wild olive-trees are included among the vegetation of the Meda Gran island. Nonetheless, the number of trees is very limited, since the plant life on the islands is influenced by the materials brought mainly by birds, especially seagulls [5].

The Mediterranean olive tree (*Olea europaea L*. subsp. *europaea*, Oleaceae) is one of the earliest fruit trees cultivated in the Mediterranean Basin. The natural distribution of the Mediterranean olive encompasses the var. *Sylvestris* (also named oleaster) and the cultivated tree var. *europaea* [6,7], some of which produce ivory-white fruit. Oleasters are usually located in isolated areas far from cultivated zones and are characterized by small fruits with a thin mesocarp [7,8]. For this reason, the biological resource monitoring provides vital information to guide conservation management and improve its effectiveness [9]. Actually, true wild forms are becoming increasingly scarce, and a few genuine wild populations have been localized in isolated areas from olive tree cultivation in Spain [10]. Wild olive-trees forests in the south of Spain can still be found, and scattered genuine wild forms in other parts such as Catalonia. [11,12]. The presence of a few wild olive trees which produce ivory-white fruits has been observed in prospecting surveys in Spain (Belaj A, unpublished results) but up to now no further attention has been paid to their study. In this sense, to the best of our knowledge, the only cultivated genotypes with ivory-white fruits are Leucocarpa in Italy [13,14], Marfil in Spain [15], and Bajda in the Maltese islands [16].

Olive oil contains bioactive components, such as monounsaturated fatty acids (55–83%), unsaponifiable compounds (1–2%), and soluble or hydrophilic compounds, including α-tocopherol, phenolic compounds, and other compounds with antioxidant properties [17]. However, most studies on wild olive genotypes deal with their diversity and only a few of them with their agronomic and oil quality evaluation [18,19]. In this sense, genotype is one of the main agronomic factors responsible for the chemical and organoleptic footprint of the olive oil strongly contributing in its qualitative and nutritional characteristics [20,21] and productivity as well [22]. On the other hand, virgin olive oil plays a crucial role in the eating habits of several dietary regimes, mainly of the Mediterranean diet [23]. The present study is part of an ongoing project related with the valorization of local cultivated and wild olive genetic resources in Catalonia (Norh-Eastern Spain). In this sense, we aimed to explore the natural habitat of the MI for the development of native oleaster olive fruit as a resource to maintain olive oil biodiversity in the Mediterranean Basin. The quality of this oil was extensively analyzed using sensory, physicochemical, and stability parameters.

## 2. Materials and Methods

### 2.1. Chemicals

Oleocanthal and oleacein were purchased from PhytoLab GmbH (Vestenbergsgreuth, Germany); oleuropein aglycone from the Toronto Research Chemical Inc. (Ontario, Canada); luteolin and nonadecanoic acid from Sigma-Aldrich (Madrid, Spain); ferulic acid and apigenin from Fluka (Buchs, Switzerland) and hydroxytyrosol from Extrasynthese (Genay, France). Hexane, methanol, acetonitrile were purchased from Sigma-Aldrich, and ciclohexane from Panreac.

### 2.2. Plant Material

The study was undertaken using plant material obtained from targeted monitoring performed in 2012 with permission from the Catalan Government. The Institute of Agrifood Research and Technology (IRTA, Constantí, Spain) and the Andalusian Institute for Research and Training in Agriculture, Fisheries, Food and Ecological Production (IFAPA, Cordoba, Spain) surveyed the wild olive population in the MI. Two oleasters with ivory-white fruit were localized. In order to maintain ex situ and monitor the natural process under controlled conditions seeds from one ivory olive tree were collected and sown in a greenhouse at the IRTA facilities. In the spring of 2014, the surviving plantlets were transplanted in the field. Cultural practices at the orchard are the usual in the producing area, and no irrigation is supplied. The orchard is sited at latitude 41.172° N and longitude 1.169° E with 100 m altitude.

### 2.3. Olive Oil Extraction

Seven out of 78 trees (12%) produced ivory-white fruits. In autumn 2018, healthy olive fruits (3–5 kg) from these trees were picked by hand and carefully stored in ventilated boxes and processed within 8 h. The physical characteristics of the fruit were measured including the ripening index (adapted to the whitish characteristic of these fruits), fruit and pit weight, moisture and oil content [24]. Shortly, fruits were classified depending on their skin and flesh color into eight categories (0—green, 1—yellow, 2—whitish areas covering less than half of the surface, 3—white color more than half of the surface, 4—white color total surface with green flesh). Average fruit and pit weight were measured using a weighing scale and flesh to pit ratio was scored. One hundred fruits were grounded in order to measure moisture content (in oven at 105 °C till constant weight) and oil content was measured by nuclear magnetic resonance (Maran-S60; Oxford Instruments, Abingdon, UK).

The washed fruits were pressed using an Abencor oil extraction system MC2 (Ingenenia y Sistemas, Sevilla, Spain), which reproduces the industrial process at a laboratory scale. The olive paste was fed into a thermomixer at 28 °C for 30 min and subsequently centrifuged at 3000 rpm (7402203.301 N) for 3 min without the addition of either warm water or talc as coadjuvant. The oil was separated by decanting, transferred into glass bottles. and then stored in the dark at −20 °C until analysis.

### 2.4. Olive Oil Characterization

#### 2.4.1. Fatty Acid Composition

Fatty acids were transmethylated with boron trifluoride (BF_3_) and methanolic potassium hydroxide [25]. Then, fatty acids were determined by gas chromatography (Agilent 6890N, Boston, USA), fitted with a flame ionization detector (FID) and a capillary column (0.25 mm × 0.25 μm × 30 m; DB23, Agilent, Bellefonte, USA). Both the detector temperatures and injector were kept at 250 °C; and the oven temperature at 170 °C. The carrier gas was helium with a flux of 55.8 mL/min, and a pressure of 10.99 psi at the column head. The injection volume was set 1 μL. The internal standard (nonadecanoic acid) was analyzed in the same experimental conditions.

#### 2.4.2. Pigments and Color

The oil color was measured according to the CIELab system under 2 degrees and DL65 illumination conditions, using a spectrophotometer (Konica-Minolta CM-3500d, Osaka, Japan). Palette colors were described using three parameters: lightness (L*, from black to white), and the chromatic components a* (from green to red), and b* (from blue to yellow). The other parameter used was chroma [C* = (2a*+2b*)/2].

Chlorophyll and carotenoid pigments were measured by using a spectrophotometer (Shimadzu UV-160A; Kyoto, Japan). The maximum absorption at 670 and 470 nm is related to the chlorophyll and carotenoid fractions, respectively. They were used for calculation of the total content of these pigments in olive oil according to the method described by Minguez-Mosquera et al. [26]. The values of the coefficients of specific extinction applied were E0 = 613 for pheophytin as a major component in the chlorophyll fraction and E0 = 2000 for lutein as a major component in the carotenoid fraction. The pigment contents were calculated as follows:

(1) $$\mathrm{Chlorophyll}\;\left(\mathrm{mg}/\mathrm{kg}\right)=\frac{A_{670}\;\times \;DF}{613\times Ws}$$

(2) $$\mathrm{Carotenoid}\;\left(\mathrm{mg}/\mathrm{kg}\right)\;=\frac{A_{470}\;\times \;DF}{2000\times Ws}$$

where *A* is the absorbance, *DF* is dilution factor, and *Ws* is the sample weight.

#### 2.4.3. Phenolic Compounds

Then of liquid–liquid extraction, the quantification and identification of individual polyphenols were carried out by liquid chromatography coupled to mass spectrometry in tandem mode (LC-MS/MS) following procedures described previously [27]. For the liquid–liquid extraction, 0.5 g of olive oil was dissolved in hexane (oil/hexane 1:2, *w/v*) in a 10 mL centrifuge tube and shaken during 1 min. Later, 2 mL of MeOH:H_2_O (8:2 *v/v*) was added to phenolic compound extraction and the samples were homogenized during 1 min. The two phases were separated by centrifugation at 3000 rpm, 4 °C during 4 min. The methanolic fraction was separated and was subjected to a second cleaning with hexane, while the remaining phenolic content in the hexane fraction was extracted adding one more time MeOH:H_2_O (8:2 *v/v*). Before all, extracts were shaken and centrifuged in using the same conditions previously detailed. Both methanolic extracts were recovered and evaporated with N_2_. Finally, the extracts were reconstituted with 800 μL of ACN and stored at −80 °C until chromatographic analysis.

An Acquity^TM^ UPLC (Waters; Milford, MA, EUA) coupled to an API 3000 triple-quadruple mass spectrometer (PE Sciex, Concord, Ontario, Canada) with a turbo ion spray source was used. The column was an Acquity UPLC^®^ BEH C18 column (2.1 × 50 mm, i.d., 1.7 µm particle size) and Acquity UPLC^®^ BEH C18 Pre-Column (2.1 × 5 mm, i.d., 1.7 µm particle size) (Waters Corporation^®^, Wexford, Ireland). The separation of oleocanthal, oleacein, ligstroside, and oleuropein aglycone was performed MeOH (A) and H_2_O (B), both with 0.1% of formic acid as mobile phases. An increasing linear gradient (*v/v*) of B was used (t (min), %B), as follows: (0, 100); (2, 100); (4.75, 46.4); (4.9, 100); (5.9, 0); (6, 100); (6.5, 100), at a constant flow rate of 0.6 mL/min, the injection volume was 5 µL, and the column temperature was 50 °C. The separation of other phenolic compounds was achieved using H_2_O with 0.2% acetic acid (A) and ACN (B) as mobile phases. An increasing linear gradient (*v/v*) of B was used (t (min), %B), as follows: (0, 5); (2.5, 5); (12.5, 40); (12.6, 100); (13.5, 100); (13.6, 5); (15, 5), at a constant flow rate of 0.4 mL/min, the injection volume was 5 µL and the column temperature was 40 °C

The ionization was achieved using electrospray interface operating in negative mode [M–H] and the compounds were monitored in the multiple monitoring mode (MRM) with the following settings: −3500 V to capillary voltage and arbitrary units for nebulizer gas and curtain gas (N2) (10 and 12, respectively); drying gas (N2) heated to 450°C. The mass spectrometry potentials are presented in the supporting information (Table S1). The system was controlled by Analyst version 1.4.2 software supplied by Applied Biosystems. The calibration curves were prepared in refined olive oil and were linear over the concentration ranges 0–20 mg/mL using hydroxytyrosol, apigenin, luteolin, oleocanthal, oleacein, oleuropein aglycone as standards.

#### 2.4.4. Oxidative Stability

Oxidative stability was evaluated by the Rancimat method [28]. Stability was expressed as the oxidation induction time (h), measured with the Rancimat 743 apparatus (Metrohm Co., Basel, Switzerland), using an oil sample of 3.0 g that was heated to 120 °C and an air flow of 20 L/h. The oxidation induction time is the time needed for an abrupt change in conductivity of an aqueous solution in which the volatile compounds resulting from oxidation of the oil were collected.

#### 2.4.5. Sensorial Analysis

A sensorial analysis was performed by the Catalan Official Olive Oil Tasting Panel according to the UE2568/91 regulations [29]. Briefly, each oil sample was tasted by eight trained tasters in groups of four samples per session (randomly presented within each group). Positive descriptors analyzed were those of the official method (fruity, bitter, pungent), joined with other complementary ones (astringent, sweet, apple, ripe fruits, green, and other secondary undertones). The intensity of each attribute was measured using a continuous scale of 10 cm, anchored at zero and open at 10. The Catalan Panel relies under ISO-17025 certification rules and tasters were previously trained in the identification and intensity measurement of each attribute.

In all experiments, the means of five experimental runs are shown. Data are presented as mean and standard deviation.

## 3. Results and Discussion

### 3.1. Physical Characterization

The physicochemical characteristics of the ivory-white olives are shown in Table 1. With ripening, the color of the olive skin changed from green to light green-yellow and finally to white and ivory. The ripening index was 3.2 ± 0.4. The olive fruits were small (0.78 ± 0.08 g), with flesh accounting for 73% (0.57 ± 0.06 g) and the remainder to the pit (0.21 ± 0.03 g). The average fat content expressed on dry basis (db) was 28.22 ± 3.91%. Oleasters are usually characterized by small fruits with a thin mesocarp [7,8]. The results of our study are similar to that of a wild species of Tunissian trees which produce fruit of only 1 g in weight with 20.6% db of fat [19,30] and in other wild species in the Southern Spain [31]. Indeed, even the Marfil variety of ivory olives has larger fruits (2.2 g) with a higher fat content (40% db) [15]. Though fruit size and fat content can change along the ripening process [32], the very low values observed, at a relative high ripe index, makes unexpectable to reach those values typical for traditional cultivated varieties. This means that possibly we are facing a real wild olive population.

### 3.2. Fatty Acid Profile

In this work, the composition of the following nine fatty acid was determined: palmitic (C16:0), palmitoleic (C16:1), margaric (17:0), stearic (C18:0), oleic (C18:1), linoleic (C18:2), linolenic (C18:3), arachidic (C20:0), and eicosenoic (C20:1) acid (Table 2). The fatty acid composition of virgin oil is strongly affected by cultivar and other agronomical factors such as fruit ripeness, crop yield, and growing medium [33]. The fatty acid profile of the wild ivory olive population from the MI showed the typical traits of oleaster, being very rich in palmitic and palmitoleic acids and poor in oleic acid compared to oil from cultivated varieties [18,34]. Moreover, the fatty acid profile was similar to that of most common virgin olive oils; although with lower polyunsaturated fatty acid concentrations (14.9%). In our samples, the monounsaturated fatty acids comprise the largest group in the olive oil (62.5%). The high content of MUFA, particularly the oleic acid, and phenolic compounds can protect the oil to oxidation [35]. Similar ivory-white cultivars (Leucocarpa and Bajda) had been previously studied without reporting the fatty acid profile [13,14,16].

In general, the SFA content in our samples (22.6%) was found in higher concentrations than in Italian olive cultivars (13.1–17.6%) [36] and in Arbequina produced in different regions of Brazil and Spain [37]. Previous studies have demonstrated that the cultivar is an important factor that affects the composition of fatty acid in olive oils [38].

The PUFA are the substrates of enzymes that generate volatile compounds responsible for oil aroma [39] but have also been found to contribute to the rancidification of oils [40]. The MUFA / PUFA ratio holds great importance because of its effects on the nutritional properties, oxidative stability, and organoleptic characteristics of olive oils [41,42]. In the olive oil analyzed, the MUFA / PUFA ratio could indicate a moderate susceptibility to oxidation attributed to that a high ratio favors the resistance to oxidative deterioration [42]. Indeed, it has been demonstrated that oils with a high content of MUFA and low PUFA and SFA values have the best oxidative stability [37]. Moreover, the C18:1 / C18:2 ratio (oleic acid/linoleic acid ratio) can be useful to characterize olive cultivars and to have a marked relationship with stability [43].

### 3.3. Pigments and Color

Table 1 shows the pigments and chromatic parameters of the examined oils. The color characteristics of the virgin olive oil obtained from the ivory cultivar showed an intense yellow color (+b* values), with a poor green color (−a* values), bright and vivid (+L*, and chroma, respectively). The color of the oil was yellowish due to a balanced ratio between chlorophyll and carotenoid content (close to 1) [44]. Similar results were previously reported for the oil from ivory-white Leucocarpa cultivar [14].

The chlorophylls and carotenoids are very common pigments and their presence in olive oil also depends on olive variety, the stage of fruits ripeness, environmental conditions, the extraction process, and storage conditions [45,46]. Pigment determination showed chlorophyll and carotenoid concentrations of 12.5 ± 4.6 and 9.2 ± 3.3 mg/kg, respectively. Different concentrations of pigments have been reported in virgin olive oil from Leucocarpa cultivar. In this sense, higher concentrations of the total chlorophylls and carotenoids were reported (23–104 and 14–30 mg/kg, respectively) [13] and at the same time lowest concentrations were also detected (2.03–5.17 and 2.77–2.79 mg/kg, respectively) [14]. Likewise, lower concentrations of these compounds were reported previously in non-ivory cultivars from Greek islands, with values between 0.08–4.45 mg/kg and 0.87–2.28 mg/kg for chlorophylls and carotenoids, respectively [46]. Similar results have been reported in olive cultivars planted in Tunisia (Arbequina, Koroneiki, Leccino, Oueslati, and Chemchali) in the chlorophylls content (7.51–15.39 mg/kg) and less concentration in carotenoids (3.02–6.32 mg/kg) [47].

### 3.4. Phenolic Compounds

The minor polar fraction in virgin oil include different subclasses, among these: secoiridoids, phenolic alcohols, phenolic acids, flavonoids, and lignans [48] which act as antioxidant and may contribute to the prevention of several human diseases [49]. Table 3 shows the total phenol and individual phenol concentrations in olive oil obtained by liquid chromatography coupled to masses. To date, several studies have shown that the phenolic composition of virgin olive oil depends on a very complex multivariate interaction between agronomic, biochemical, and technological factors. Initially, the genetic origin of the olive, the geographical area of cultivation, the climate, and agronomic practices such as fertilization and water availability are determining factors in the composition of the olive [27,50]. Given the biological importance of these compounds, one of the purposes of this research was the phenolic profile characterization, for the first time, in ivory-white wild olive trees of Spain. As shown in Table 3, the total polyphenols were 669.97 ± 119.38 mg/kg and was higher than that obtained in oil from other wild olives (Algerian variety 242 ± 19 to 341 ± 58 mg/kg) [51].

Secoiridoids were by far the most abundant group of phenolic compounds (93%) in agreement with previous studies [52]. Oleacein (373.29 ± 72.02 mg/kg) was the main compound followed by oleocanthal (204.84 ± 52.58 mg/kg). Both compounds are responsible for the bitter and pungent test, respectively, of olive oil [53]. Such very high secoiridoids contents are really interesting from the health point of view, due to its anti-inflammatory activity that can be relevant in many pathologies [53,54]. To date, there is little information on the phenolic profile of virgin olive of ivory-white olives. In a previous study carried out with Bajda cultivar, oleacein, oleocanthal, oleuropein aglycone, and ligstroside aglycone were only identified but not quantified [16]. Leucocarpa cultivar also was analyzed and low concentrations of oleuropein aglycone were detected in the fruit (6.63 ± 0.22 to 16.8 ± 0.07 mg/g of fresh weight) whereas other compounds were not analyzed [13]. Concerning non-ivory-white cultivars, different concentrations of OLE have been reported previously in monovarietal commercial samples from Greece, reaching a maximum of 291.7 ± 10.2 mg/kg [55]. Moreover, Pendolino and Kalamon cultivars have been reported as very rich in OLE as well, with a maximum of 903 ± 394 and 866 ± 285 mg/kg, respectively [56]. Regarding OLC, high concentrations were found in Alfafara, Kalamon, and Plementa Bjelica (1602 ± 688 to 2931 ± 26 mg/kg) [56]. Similar results to our white progenies were obtained in Koroneiki cultivar (183.9 ± 5.0 to 355.0 ± 12.1 mg/kg) [55].

The hydroxytyrosol, an essential quality biomarker in virgin olive oil, was also identified in olive samples (0.77 ± 0.57 mg/kg). This phenolic alcohol was also identified in olive oil from ivory-whites [16]. The variations in the phenolic alcohols is also attributed to ripening and varietal factors [57]. Olive oil is in between the European Food Safety Authority (EFSA) parameters for claiming that polyphenols from olive oil and olive leaves protect low-density lipoproteins from oxidative damage when consumed at a daily rate of 5 mg of hydroxytyrosol and its derivatives (e.g., oleuropein complex and tyrosol) [58].

Two main flavonoids were quantified for the first time in virgin oil from wild ivory-white olive trees: luteolin (18.76 ± 10.77 mg/kg) and apigenin (1.56 ± 0.88 mg/kg). These flavonoids have been quantified in an Algerian wild olive (not ivory) but with a lower luteolin (0.97 ± 0.17 to 13 ± 3 mg/kg) and apigenin content (0.07 ± 0.01 to 1.1 ± 0.1 mg/kg) [51]. These two compounds were also identified in Bajda cultivar [16]. The Leucocarpa cultivar also produces ivory-white fruit due to low or null accumulation of flavonoid compounds [13] which is suggestive of purity and has led to the use of these olives to produce holy anointing oil [14] and with potential use as table olives [59]. The Marfil cultivar presents tolerance to olive leaf spot disease and also produces ivory-colored fruits with high oil quality [15]. In fact, the particular color of the ripe fruit of these cultivars is due to a blockage of flavonoid biosynthesis [60], mainly anthocyanins [13]. In previous studies, the content of luteolin found in Picual and Hojiblanca oils (41.3 and 22.69 ± 5.09 mg/kg, respectively) was higher to those found in the present assay. In the same studies, higher concentrations of apigenin were reported reaching concentrations to the 8.72 and 5.51 ± 0.69 mg/kg, respectively [27,61]. Differences in the concentration of both compounds in non-ivory cultivars growing in an identical climate/environment area in Italy also was reported with concentrations between 2.2 ± 0.5–16.7 ± 0.6 mg/kg for the luteolin and 0.2 ± 0.1–9.2 ± 0.3 mg/kg for the apigenin [62]. The ripening stage is another factor that affects the concentrations of these compounds, increasing the content of luteolin with the maturity index whereas apigenin does not show any definite trend. Moreover, during oil extraction the flavonoids are hardly affected by time and temperature of malaxation [61].

Other compounds were also identified in concentrations lower than 1 mg/kg (hydroxyelenolic acid and hydroxydecarboxymethyl-oleuropein aglycone, HDCM-OA).

### 3.5. Oxidative Stability

Table 1 also shows the oxidative stability measured by the Rancimat method. Oxidative stability is an important parameter for evaluating the quality of oils and fats, providing a good estimation of susceptibility to oxidative degeneration, the main cause of alterations in olive oil [63]. The oxidative stability for the ivory-white oil genotype measured by the Rancimat method was 15.8 h, which is quite a high value and is higher than that of the Marfil olive oils (12.59 h) [15,64]. High oxidative stability may be due to the equilibrium among the fatty acid profile, and the carotenoid and phenolic compounds (Table 2 and Table 3). The chlorophyll/carotenoid ratio is low. In the presence of light, chlorophylls and their derivatives are the most active promoters of photosensitized oxidation in virgin olive oil contributing greatly to a high susceptibility to oxidation [65]. Carotenoids possess conjugated hydrocarbons which are potent protectors against photosensitized oxidation, acting as singlet oxygen quencher [66]. Phenolic compounds have also been correlated with oil shelf life due to the antioxidant properties of mainly oleacein and oleuropein aglycone, which show remarkable resistance to oxidation [67]. Finally, the low content of polyunsaturated fatty acids (14.9%), which are the fatty acids most prone to oxidation, also contributed to oxidative stability [68].

### 3.6. Sensorial Analysis

**Figure 1:**
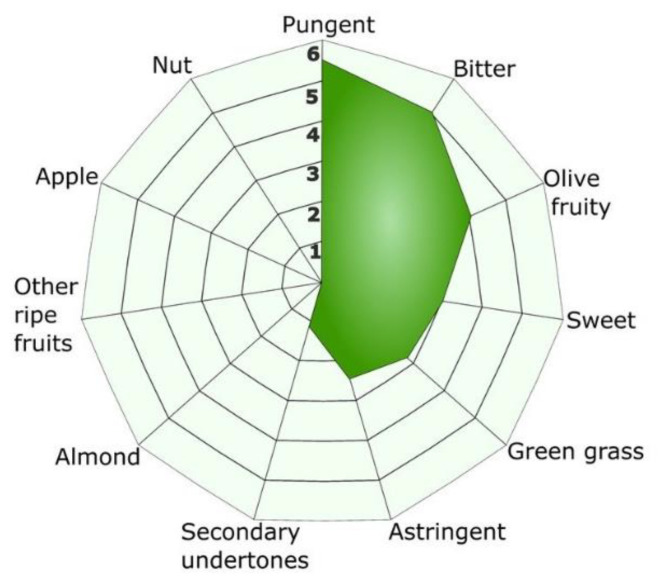
Sensory virgin olive oil characteristics.

Sensorial analysis resulted in medium to high intensity fruitiness (5.0 ± 0.3), clearly pungent (5.5 ± 0.3) and bitter (5.0 ± 0.7). Secondary attributes were also detected such as medium intensity sweetness, cut grass aroma, and slight final almond and astringent mouthfeel. Neither ripe fruit nor apple attributes were detected. The sensory parameters of the oil of the ivory-white genotype revealed the complexity of olive oil. The taste has fruity, bitter, and pungent characteristics, the latter two sensations being generated in the same gustative papillae [69,70]. Two phenolic compounds, oleocanthal and oleacein, that are exclusively present in virgin olive oil, seem to be the main compounds responsible for these mouthfeel perceptions and were the major phenolic compounds found in the olive oil from the ivory-white cultivar. Volatile compounds are responsible for the aggregate aroma of virgin olive oil. The fruity notes are perceived directly and/or through the back of the nose and are the characteristic set of olfactory sensations of the oil from sound, fresh olives, either ripe or unripe [71]. Fruity notes are mainly given by volatile compounds produced by the lipoxygenase pathway during oil extraction. Green flavor, and sweet, grassy, and almond aromas [72] have been described in olives of ivory-white progeny (Figure 1).

## 4. Conclusions

Actions to promote the conservation of indigenous oleaster genotypes will prevent their decline at regional, national, and global levels. Native wild olive trees are becoming increasingly scarce, and thus, protected reserve zones are fundamental pillars for the preservation of these wild species which have adapted to a series of abiotic and biotic factors. At least, two wild olive-tree were localized in the natural reserve of the MI what produces ivory-white olive fruits. Physicochemical characterization of these fruits and olive oil revealed unique sensory physicochemical and stability characteristics, highlighting the need to maintain the biodiversity of olive oil and food authenticity of this traditional Mediterranean food. However, further research should be performed in this case on sensory characteristics to determine its possible applications as an edible oil for human nutrition. Nevertheless, additional studies during several years and involving more oleasters are crucial to corroborate the results obtained and to better characterize wild olive genetic resources in Catalonia.

## Figures and Tables

**Table 1:** Physical and chemical characterization of olive oil from ivory-white olives.

Physical Characteristics		Chromatic Parameters		Pigments (mg/kg)	
Fruit weight (g)	0.78 ± 0.08	L*	88.6 ± 6.7	Chlorophylls	12.5 ± 4.6
Flesh weight (g)	0.57 ± 0.06	a*	−9.3 ± 1.4	Carotenoids	9.2 ± 3.3
Pit weight (g)	0.21 ± 0.03	b*	83.7 ± 23.3	Chlorophylls/carotenoids	1.4 ± 1.4
Fat content (%)	28.22 ± 3.91	Chroma	84.2 ± 23.3	**Oxidative Stability (h at 120 °C)**	
Ripening index	3.18 ± 0.42	a*/b*	−0.1 ± 0.06	Stability	15.8 ± 5.9

**Table 2:** Physical and chemical characterization of ivory-white olives.

Fatty Acid Composition (%)			
Palmitic	20.11 ± 2.46	Fatty acid indices	
Palmitoleic	3.51 ± 0.96	MUFA (%)	62.51 ± 6.46
Margaric	0.39 ± 0.37	PUFA (%)	14.90 ± 4.20
Stearic	1.79 ± 0.07	MUFA / PUFA	4.20 ± 2.60
Oleic	58.80 ± 7.05	C18:1 / C18:2	4.36 ± 3.16
Linoleic	13.48 ± 4.09	SFA (%)	22.60 ± 2.48
Linolenic	1.28 ± 0.18	UFA / SFA	3.43 ± 0.53
Arachidic	0.30 ± 0.02		
Eicosenoic	0.20 ± 0.04		
Eicosadienoic	0.14 ± 0.13		

SFA saturated fatty acids, PUFA polyunsaturated fatty acids, MUFA monounsaturated fatty acid, UFA unsaturated fatty acids.

**Table 3:** Phenolic compound profile of olive oil from ivory-white olives.

Phenolic Compounds (mg/kg)	
Total phenols	669.97 ± 119.38
Oleacein	373.29 ± 72.02
Oleocanthal	204.84 ± 52.58
Oleuropein aglycone	33.82 ± 12.61
Ligstroside aglycone	14.15 ± 3.34
Elenolic acid	17.18 ± 9.47
Hydroxytyrosol	0.77 ± 0.57
Hydroxyelenolic acid	0.27 ± 0.19
Lactone	4.94 ± 1.77
HDCM-OA	0.37 ± 0.18
Luteolin	18.76 ± 10.77
Apigenin	1.56 ± 0.88

HDCM-OA hydroxydecarboxymethyl-oleuropein aglycone.

## Supplementary Materials

The following are available online at https://www.mdpi.com/2076-3921/9/10/1009/s1, Table S1: Multiple reaction monitoring conditions for the polyphenols.
