# Supplementary material for: Conservation of Native Wild Ivory-White Olives from the MEDES Islands Natural Reserve to Maintain Virgin Olive Oil Diversity
Source: Antioxidants (Basel). 2020 Oct 17;9(10):1009. doi: 10.3390/antiox9101009 (PMC7603032; doi:10.3390/antiox9101009)
Supplement: Supplementary file 1 [file antioxidants-09-01009-s001.pdf]

Table S1 Multiple reaction monitoring conditions for the polyphenols

**Phenolic compounds**	**m/z**	**DP**	**FP**	**EP**	**CE**	**RT**
Oleuropein A	377/241	-30	-140	-10	-30	9.14
HDCM OA	335/199	-30	-140	-10	-30	7.32
Lactone	321/185	-40	-250	-10	-20	7.16
Ligstroside A	361/291	-30	-140	-10	-30	9.01
Oleocanthal	303/285	-40	-170	-5	-10	5.92
Elenolic acid	241/127	-30	-140	-10	-30	6.31
Hydroxyelenolic acid	257/137	-30	-140	-10	-30	6.02
Luteolin	285/133	-100	-340	-10	-50	8.15
Apigenin	269/117	-70	-200	-10	-50	9.11
Hydroxytyrosol	153/123	-40	-250	-10	-20	1.13

DP: declustering potential; FP: focusing potential; EP: entrance potential; CE: collision energy; RT: retention time
